# Increased serum asprosin is correlated with diabetic nephropathy

**DOI:** 10.1186/s13098-021-00668-x

**Published:** 2021-05-01

**Authors:** Rui Wang, Peng Lin, Huibo Sun, Wenchao Hu

**Affiliations:** 1 Department of Blood Transfusion, Qilu Hospital (Qingdao), Cheeloo College of Medicine, Shandong University, Qingdao, People’s Republic of China; 2 Department of Endocrinology, Qilu Hospital, Cheeloo College of Medicine, Shandong University, Jinan, People’s Republic of China; 3 Department of Endocrinology, Qilu Hospital (Qingdao), Cheeloo College of Medicine, Shandong University, 758 Hefei Road, Shibei District, Qingdao, 266035 Shandong China

**Keywords:** Asprosin, Diabetic nephropathy, Renal function, Urine albumin to creatinine ratio

## Abstract

**Objective:**

The adipokine asprosin, which was recently discovered, facilitates hepatic glucose production. The aim of this study is to see whether serum asprosin concentrations are linked to diabetic nephropathy (DN).

**Methods:**

We performed this investigation in a group of 212 type 2 diabetes (T2DM) patients. These patients were classified into three subgroups: DN0 group (normal to mildly increased), DN1 group (moderately increased), and DN2 group (severely increased) on the basis of urine albumin-to-creatinine ratio (ACR).

**Results:**

When compared to the controls, T2DM patients had higher serum asprosin levels. The DN2 group had significantly higher serum asprosin than the DN0 and DN1 groups. Furthermore, the DN1 group had higher serum asprosin than the DN0 group. Serum asprosin was linked to a higher risk of T2DM and DN in a logistic regression analysis. Serum asprosin was found to be positively related with disease duration, systolic blood pressure, blood urea nitrogen, creatinine, uric acid, ACR, calcium channel blockers, and angiotensin-converting enzyme inhibitor/angiotensin II receptor blocker therapy, but negatively related with glomerular filtration rate, metformin, and acarbose therapy.

**Conclusion:**

Serum asprosin increase with the progression of DN. Serum asprosin is correlated with renal function and ACR.

## Introduction

Diabetes mellitus is becoming more common around the world [1]. Diabetic nephropathy (DN), the most frequent diabetic complication, primarily caused the result of end-stage renal disease (ESRD) [2]. DN is responsible for 25–35 percent of type 2 diabetes mellitus (T2DM) cases and significantly raises T2DM patient mortality [3]. The underlying mechanism of DN is currently being investigated. However, the precise mechanism of DN is still uncertain [4]. Serum biomarkers, such as glycated albumin, copeptin, cystatin C, 8-hydroxy-2′-deoxyguanine have been recently utilized to diagnose DN or assess the progression of DN [5–8]. Investigating new predictive biomarkers will provide an opportunity for preventive or therapeutic interventions to prevent or delay the occurrence of ESRD.

Asprosin, a C-terminal cleavage peptide developed by profibrillin [9], is a recently found adipokine. Asprosin is released from white adipose and transferred to the liver. Asprosin causes hepatic gluconeogenesis and results in increased circulating insulin and glucose concentrations [9]. Plasma asprosin permeates the blood–brain barrier and stimulates orexigenic neurons directly through a cAMP-dependent signal, triggering appetite stimulation and weight gain [10]. In humans, a genetic mutation of asprosin causes a condition characterized by a lack of appetite and excessive leanness [10].

Recent research has found a relation between serum asprosin and diabetes. Serum asprosin are substantially higher in T2DM subjects than in controls [11–13]. Furthermore, plasma asprosin levels are substantially higher in the impaired glucose tolerance compared with normal controls [14]. Criculating asprosin has also been demonstrated to be related with fasting blood glucose and glycosylated hemoglobin [12, 14]. However, no prior research has looked into the relation between asprosin and diabetic complications. As a result, we expect to test the hypothesis that serum asprosin levels are linked to DN.

## Materials and methods

### Patients

This investigation was conducted in 212 T2DM patients who were enrolled from the inpatient Department of Endocrinology of Qilu Hospital. These subjects were then classified into three groups based on the measurements of the urinary albumin to creatinine ratio (ACR) [15]: DN0 (normal to mildly increased, ACR < 30 mg/g, 94 subjects), DN1 (moderately increased, 30 ≤ ACR ≤ 300 mg/g, 82 subjects), and DN2 (severely increased, ACR > 300 mg/g, 36 subjects).

The following were the exclusion criteria: type 1 diabetes, cancer, and acute infection during the preceding 3 months. The control group consisted of 72 healthy adults. They performed an oral glucose tolerance test and found that it was normal.

The hospital ethics board approved this report, and it was performed in accordance with the Declaration of Helsinki.

### Measurements

Anthropometric information including height, weight, blood pressure, and therapy information such as anti-hypertensive drugs, statins, and anti-diabetic drugs were recorded. The body mass index (BMI) was determined a formula as weight/height^2^. The Cockroft–Gault formula was used to measure the glomerular filtration rate (GFR).

A biochemistry automated analyzer (Hitachi 7170, Tokyo, Japan) was used to measure blood lipids, renal function parameters. High-performance liquid chromatography was used to evaluate HbA1c levels. The ACR was assessed three times and the average results were used. A commercial ELISA kit was used to test serum samples for asprosin (Phoenix Pharmaceuticals, Inc, USA). The results were checked in duplicate.

### Statistical analysis

The data is viewed as the forms of means ± standard deviations or median (interquartile). ANOVA, Chi-square, and Kruskal–Wallis test were used to assess variable differences between groups. Logistic regression analysis was conducted to evaluate the variables related with T2DM and DN. Linear regression analysis was used to evaluate the relationship between serum asprosin and other variables. *P* < 0.05 was set as statistical significant.

## Results

### Baseline parameter comparison

Systolic blood pressure (SBP), HbA1c were higher, while high-density lipoprotein cholesterol (HDL-C) was lower in the case group compared to controls (Table 1). The DN2 group also had substantially higher SBP, blood urea nitrogen (BUN), creatinine (Cr), and uric acid (UA) as well as lower GFR compared to DN1, DN0, and control groups (Table 1).

**Table 1:** Clinical characteristic of T2DM patients and controls

	Control	T2DM patients			
		DN0	DN1	DN2	*p*
N	72	94	82	36	
Age (years)	58.42 ± 8.01	57.55 ± 11.57	58.39 ± 13.22	59.78 ± 11.32	0.791
Gender (M/F)	41/31	51/43	41/41	19/17	0.855
Duration (years)	–	8.53 ± 1.68	10.15 ± 2.43^b^	12.06 ± 2.34^bc^	< 0.001
BMI (kg/m^2^)	25.88 ± 2.85	26.25 ± 4.05	26.16 ± 3.5	26.32 ± 3.1	0.901
SBP (mmHg)	122.01 ± 11.48	135.8 ± 22.39^a^	146.71 ± 30.57^ab^	156.69 ± 22.51^abc^	< 0.001
DBP (mmHg)	78.96 ± 7.89	80.85 ± 14.75	87.56 ± 20.22^ab^	85.69 ± 12.83^ab^	0.002
HbA1c (%)	4.75 ± 0.31	7.82 ± 1.48^a^	8.1 ± 1.21^a^	7.76 ± 1.48^a^	< 0.001
TG (mmol/L)	2.04 ± 1.59	1.88 ± 1.14	2.04 ± 1.65	2.13 ± 1.12	0.775
TC (mmol/L)	5.28 ± 0.91	5 ± 0.98	5.41 ± 1.2	5.42 ± 1.02^b^	0.041
HDL-C (mmol/L)	1.5 ± 0.24	1.11 ± 0.23^a^	1.13 ± 0.21^a^	1.16 ± 0.33^a^	< 0.001
LDL-C (mmol/L)	3.28 ± 0.54	3.33 ± 0.81	3.65 ± 0.99^ab^	3.61 ± 0.8^a^	0.008
BUN (mmol/L)	5.42 ± 1.18	5.36 ± 1.64	5.9 ± 1.97	8.85 ± 4.1^abc^	< 0.001
Cr (μmol/L)	66.6 ± 10.65	66.32 ± 18.91	65.95 ± 21.12	120.75 ± 72.65^abc^	< 0.001
UA (μmol/L)	305.11 ± 70.95	301.42 ± 87.54	296.39 ± 76.9	356.22 ± 88.31^abc^	0.002
ACR (mg/g)	–	16.14 ± 4.48	96.08 ± 83.67^b^	> 300^bc^	< 0.001
GFR (mL/min/1.73 m^2^)	101.66 ± 12.09	109.26 ± 41.65	109.14 ± 33.24	67.46 ± 35.53^abc^	< 0.001
Asprosin (ng/mL)	11.86 (9.17–14.56)	15.43 (12.95–18.26)^a^	17.38 (13.86–19.56)^ab^	22.52 (20.25–24.58)^abc^	< 0.001
Treatment					
Metformin (n, %)	–	68 (72.3%)	58 (70.7%)	19 (52.8%)	0.084
Acarbose (n, %)	–	46 (48.9%)	44 (53.7%)	13 (36.1%)	0.213
Sulfonylureas (n, %)	–	60 (63.8%)	52 (63.4%)	21 (58.3%)	0.834
DPP-IV inhibitor (n, %)		46 (48.9%)	36 (43.9%)	15 (41.7%)	0.691
Insulin (n, %)	–	37 (39.4%)	26 (31.7%)	17 (47.2%)	0.252
Statin (n, %)	–	47 (50%)	45 (54.9%)	21 (58.3%)	0.651
CCB (n, %)	–	51 (54.3%)	59 (72%)^b^	31 (86.1%)^bc^	0.001
ACEI/ARB (n, %)	–	45 (47.9%)	55 (67.1%)^b^	32 (88.9%)^bc^	< 0.001

*T2DM* type 2 diabetes mellitus, *BMI* body mass index, *SBP* systolic blood pressure, *DBP* diastolic blood pressure, *TG* triglycerides, *TC* total cholesterol, *HDL-C* high-density lipoprotein cholesterol, *LDL-C* low-density lipoprotein cholesterol, *BUN* blood urea nitrogen, *Cr* creatinine, *UA* uric acid, *ACR* urine albumin to creatinine ratio, *GFR* glomerular filtration rate, *DPP-IV inhibitor* dipeptidyl peptidase IV inhibitor, *CCB* calcium channel blockers, *ACEI* angiotensin-converting enzyme inhibitor, *ARB* angiotensin II receptor blockers

^a^Significant versus control subjects

^b^Significant versus  DN0 group

^c^Significant versus  DN1 group

As displayed in Table 1, the DN2 and DN1 subjects had longer disease duration, blood pressure, higher proportion of angiotensin-converting enzyme inhibitor (ACEI)/angiotensin II receptor blockers (ARB) and calcium channel blockers (CCB) therapy than DN0 subjects. Furthermore, the DN2 subjects had longer disease duration and a higher proportion of CCB and ACEI/ARB therapy than the DN1 subjects.

### Serum asprosin differences

Table 1 shows that when compared to the control group, all T2DM patients had substantially higher serum asprosin. When compared to the DN1 and DN0 subjects, the DN2 subjects had substantially higher serum asprosin. The DN1 subjects also had substantially higher serum asprosin than the DN0 subjects.

### Relation between serum asprosin and T2DM

T2DM group had substantially higher serum asprosin than the control group [17.06 (13.92–19.57) ng/mL vs. 11.86 (9.17–14.56) ng/mL, *P* < 0.001]. Table 2 shows that SBP, diastolic blood pressure (DBP), HDL-C, BUN, and serum asprosin were all related to T2DM in a univariate logistic regression model. Serum asprosin was still related to T2DM after adjusting the confounding factors.

**Table 2:** Logistic regression analysis for determining the factors associated with T2DM

Characteristics	Univariate logistic regression		Multiple logistic regression	
	OR (95% CI)	*P*	OR (95% CI)	*P*
Age (years)	0.999 (0.975–1.023)	0.916	–	–
Gender (M/F)	1.203 (0.702–2.063)	0.501	–	–
BMI (kg/m^2^)	1.029 (0.952–1.113)	0.47	–	–
SBP (mmHg)	1.055 (1.036–1.075)	< 0.001	1.112 (1.06–1.168)	< 0.001
DBP (mmHg)	1.029 (1.006–1.051)	0.012	1.021 (1.002–1.043)	0.016
TG (mmol/L)	0.974 (0.811–1.171)	0.782	–	–
TC (mmol/L)	0.955 (0.741–1.231)	0.723	–	–
HDL-C (mmol/L)	0.004 (0.001–0.016)	< 0.001	0.002 (0.001–0.017)	< 0.001
LDL-C (mmol/L)	1.415 (0.996–2.009)	0.052	–	–
BUN (nmol/L)	1.189 (1.023–1.382)	0.024	1.087 (0.821–1.441)	0.56
Cr (μmol/L)	1.011 (0.999–1.023)	0.08	–	–
UA (μmol/L)	1.001 (0.997–1.004)	0.743	–	–
GFR (mL/min/1.73 m^2^)	1 (0.993–1.008)	0.924	–	–
Asprosin (ng/mL)	1.623 (1.428–1.843)	< 0.001	1.635 (1.373–1.947)	< 0.001

Abbreviation as Table 1

### Relation between serum asprosin and DN

T2DM groups with moderately and severely increased ACR were considered as the DN group. DN group had substantially higher asprosin than non-DN group [18.3 (15.01–20.55) ng/mL vs. 15.43 (12.95–18.26) ng/mL, *P* < 0.001]. Table 3 shows disease duration, SBP, DBP, low-density lipoprotein cholesterol (LDL-C), Total cholesterol (TC), BUN, Cr, GFR, serum asprosin, CCB, ACEI/ARB therapy were all related to DN in a univariate logistic regression model. Serum asprosin was still related to DN after adjusting the confounding factors (Table 3).

**Table 3:** Logistic regression analysis for determining the factors associated with DN

Characteristics	Univariate logistic regression		Multiple logistic regression	
	OR (95% CI)	*P*	OR (95% CI)	*P*
Age (years)	1.009 (0.986–1.031)	0.453	–	–
Gender (M/F)	1.147 (0.666–1.973)	0.622	–	–
Duration (years)	1.672 (1.404–1.991)	< 0.001	1.599 (1.318–1.941)	< 0.001
BMI (kg/m^2^)	0.997 (0.926–1.074)	0.94	–	–
SBP (mmHg)	1.022 (1.01–1.034)	< 0.001	1.012 (0.988–1.036)	0.332
DBP (mmHg)	1.024 (1.006–1.043)	0.011	1.008 (0.98–1.038)	0.572
HbA1c (%)	1.094 (0.898–1.333)	0.372	–	–
TG (mmol/L)	1.116 (0.9–1.384)	0.316	–	–
TC (mmol/L)	1.451 (1.104–1.907)	0.008	1.845 (0.581–5.861)	0.299
HDL-C (mmol/L)	1.782 (0.575–5.519)	0.317	–	–
LDL-C (mmol/L)	1.525 (1.098–2.118)	0.012	0.659 (0.163–2.668)	0.559
BUN (nmol/L)	1.326 (1.142–1.539)	< 0.001	1.301 (1.02–1.661)	0.034
Cr (μmol/L)	1.015 (1.004–1.026)	0.008	0.995 (0.974–1.016)	0.639
UA (μmol/L)	1.002 (0.999–1.005)	0.267	–	–
GFR (mL/min/1.73 m^2^)	0.991 (0.984–0.999)	0.028	1.005 (0.993–1.017)	0.397
Asprosin (ng/mL)	1.242 (1.142–1.35)	< 0.001	1.183 (1.064–1.315)	0.002
Treatment				
Metformin (n, %)	0.718 (0.398–1.295)	0.271	–	–
Acarbose (n, %)	0.975 (0.567–1.677)	0.927	–	–
Sulfonylureas (n, %)	0.919 (0.524–1.611)	0.769	–	–
DPP-IV inhibitor (n, %)	0.794 (0.461–1.369)	0.407	–	–
Insulin (n, %)	0.883 (0.505–1.544)	0.663	–	–
Statin (n, %)	1.269 (0.737–2.186)	0.39	–	–
CCB (n, %)	2.71 (1.507–4.874)	0.001	1.023 (0.382–2.736)	0.964
ACEI/ARB (n, %)	3.056 (1.718–5.436)	< 0.001	1.348 (0.508–3.573)	0.549

Abbreviation as Table 1

### Correlation of serum asprosin with other variables

After a simple linear regression study, serum asprosin was related to disease duration, SBP, ACR, BUN, Cr, UA, GFR, metformin, acarbose, CCB, and ACEI/ARB therapy (Table 4). After multiple linear regression analysis, BUN and ACR remained to be related to serum asprosin.

**Table 4:** The correlation between serum asprosin concentrations and various parameters in T2DM patients

Parameters	Simple regression analysis		Multiple regression analysis	
	R	*P*	β	*P*
Age (years)	0.066	0.338		
Gender (M/F)	0.015	0.824		
Duration (years)	0.235	0.001	0.023	0.746
BMI (Kg/m^2^)	0.034	0.619		
SBP (mmHg)	0.22	0.001	0.087	0.302
DBP (mmHg)	0.07	0.31		
HbA1c (%)	0.065	0.35		
TG (mmol/L)	0.05	0.472		
TC (mmol/L)	0.043	0.533		
HDL-C (mmol/L)	-0.007	0.917		
LDL-C (mmol/L)	0.032	0.644		
BUN (nmol/L)	0.404	< 0.001	0.219	0.03
Cr (μmol/L)	0.304	< 0.001	0.159	0.158
UA (μmol/L)	0.163	0.018	0.09	0.21
ACR (mg/g)	0.466	< 0.001	0.323	< 0.001
GFR (mL/min/1.73 m^2^)	− 0.283	< 0.001	0.078	0.356
Treatment				
Metformin (n, %)	− 0.143	0.037	− 0.009	0.907
Acarbose (n, %)	− 0.148	0.031	− 0.08	0.282
Sulfonylureas (n, %)	− 0.121	0.078		
DPP-IV inhibitor (n, %)	− 0.072	0.295		
Insulin (n, %)	0.079	0.255		
Statin (n, %)	0.009	0.897		
CCB (n, %)	0.163	0.018	− 0.002	0.997
ACEI/ARB (n, %)	0.23	0.001	0.069	0.423

Abbreviation as Table 1

## Discussion

Asprosin, a recently found adipokine, increase circulating glucose and insulin levels by promoting hepatocyte glucose production and output [9]. Lee et al. reported that asprosin incubation promotes the apoptosis and dysfunction of insulinoma cells and human islets through a toll-like receptor 4 (TLR4) /JNK-mediated pathway [16], suggesting that asprosin causes β cell dysfunction. Furthermore, asprosin exacerbates insulin sensitivity which is assessed using glucose utilization, and insulin receptor substrate 1 and Akt phosphorylation levels [17]. The asprosin receptor OLFR734 modulates hepatic glucose output [18]. OLFR734^−/−^ mice exhibits reduced hepatic glucose output and increased insulin sensitivity [18]. Asprosin and its receptor OLFR734 might be used as a new mechanism for the T2DM therapy.

Recent study reported that asprosin levels increase at 24 h after coronary angiography compared with those at the time of admission because of unstable angina pectoris [19]. Changes in asprosin levels are positively correlated with syntax scores which are utilized to assess disease severity [19]. The focus of this study was on macrovascular disease. Asprosin can be related to diabetic macrovascular complications according to this report. Our findings revealed a relation between elevated serum asprosin concentrations and DN. Another investigation showed that serum asprosin is independently associated with ACR in T2DM population [20]. Circulating asprosin may be used as a novel serum biomarker for DN evaluation.

Asprosin incubation promotes inflammation reaction via the TLR4/JNK-mediated signal pathway in insulinoma cells and human islets [13]. Asprosin treatment also induces inflammation markers including NF-κβ, interleukin-6, and phosphorylated IκB [14]. These findings revealed a strong relation between asprosin and inflammation. Low-grade inflammation is characterized as an innate immune system reaction that has been activated [21]. Increased circulating pro-inflammatory cytokines that stimulate the immune system are clinically identified as this form of inflammation. Chronic inflammation is related to the mechanism of T2DM and DN [22]. As a result, asprosin is thought to play a role in DN through regulating inflammatory response.

There are a few limitations in this report. First, the claim is challenged by the small sample size. Second, the data’s cross-sectional design limits the conclusion's intensity. Future longitudinal research would be required to validate the causal relationship. Last, the healthy controls were recruited from the subjects for health check-up. They all did OGTT to exclude T2DM upon their own request. Therefore, they may have some risk factors for diabetes or worry about developing diabetes. This possibility might have introduced bias to the final results.

## Conclusions

In conclusion, as the disease progresses, serum asprosin rise with the progression of DN. Serum asprosin is related to renal function and ACR. In addition to conventional approaches for assessing the risk and progression of DN, serum asprosin may be used as a potential serum biomarker.

## Data Availability

Data are available upon reasonable request.
